# Molecular Identification of Endophytic Fungi and Their Pathogenicity Evaluation Against *Dendrobium nobile* and *Dendrobium officinale*

**DOI:** 10.3390/ijms21010316

**Published:** 2020-01-02

**Authors:** Surendra Sarsaiya, Archana Jain, Qi Jia, Xiaokuan Fan, Fuxing Shu, Zhongwen Chen, Qinian Zhou, Jingshan Shi, Jishuang Chen

**Affiliations:** 1 Key Laboratory of Basic Pharmacology and Joint International Research Laboratory of Ethnomedicine of Ministry of Education, Zunyi Medical University, Zunyi 563003, China; sarsaiya.s@gmail.com (S.S.); assjain80@gmail.com (A.J.); 2 Bioresource Institute for Healthy Utilization, Zunyi Medical University, Zunyi 563003, China; jiaqi_1003@163.com (Q.J.); ncusfx@163.com (F.S.); 15195884364@163.com (Z.C.); zhouqnzmu@163.com (Q.Z.); 3 College of Biotechnology and Pharmaceutical Engineering, Nanjing Tech University, Nanjing 211800, China; biofanxk@163.com

**Keywords:** Dendrobium, molecular identification, endophytic fungi, pathogenicity, protocorm, seedling

## Abstract

*Dendrobium* are tropical orchid plants that host diverse endophytic fungi. The role of these fungi is not currently well understood in *Dendrobium* plants. We morphologically and molecularly identified these fungal endophytes, and created an efficient system for evaluating the pathogenicity and symptoms of endophytic fungi on *Dendrobium nobile* and *Dendrobium officinale* though in vitro co-culturing. ReThe colony morphological traits of *Dendrobium* myco-endophytes (DMEs) were recorded for their identification. Molecular identification revealed the presence of *Colletotrichum tropicicola*, *Fusarium keratoplasticum*, *Fusarium oxysporum*, *Fusarium solani*, and *Trichoderma longibrachiatum*. The pathogenicity results revealed that *T. longibrachiatum* produced the least pathogenic effects against *D. nobile* protocorms. In seedlings, *T. longibrachiatum* showed the least pathogenic effects against *D. officinale* seedlings after seven days. *C. tropicicola* produced highly pathogenic effects against both *Dendrobium* seedlings. The results of histological examination of infected tissues revealed that *F. keratoplasticum* and *T. longibrachiatum* fulfill Koch’s postulates for the existence of endophytes inside the living tissues. The DMEs are cross-transmitted inside the host plant cells, playing an important role in plant host development, resistance, and alkaloids stimulation.

## 1. Introduction

The Orchidaceae is the chief family of plants, with over 25,000 plant species worldwide. They are also some of the most vulnerable flowering plants, as numerous genera are endangered and nearly all genera are at risk of habitat harm and over-assortment [1]. China is a rich source of orchid plants, with over 1447 species, mostly located in the subtropical and tropical provinces in the southwest and south [1,2]. Many *Dendrobium* orchids are horticultural plants and have been used for profitable trade due to their flowering profusion, extensive variety of flower colors, shapes, sizes, and year-round producibility, along with long lifespan [3]. Most species are in danger of extinction. Orchids extend their lives as herbaceous plants using two evolutionary methods: sympodial growth and monopodial development, which are influenced by the many endophytic fungal species as main pathogens of orchids [4,5]. *Dendrobium officinale* has been extensively used in traditional medicines for over 2000 years to decrease fever, inhibit tumors, increase antioxidant activity, treat hypoglycemia, recover from loss of eyesight, and control the immune system, according to the best of China Pharmacopeia [6,7].

*Dendrobium* plants parts, for example, roots, stems, buds, and leaves of tropical orchid, harbor diverse fungal taxa, including mutualistic mycorrhiza, and endophytic fungi and considerably diverse nonmycorrhizal fungal associates. The role of the root-allied fungi is not well understood. They typically originate in the velamen, without causing any disease symptoms [8]. They may encourage the growth of *Dendrobium* by activating soil chemicals in the rhizosphere. The impact of quantities or variations of secondary metabolite have been investigated. At large, they act as a supply for bioactive molecules that defend the host from rhizospheric pathogens [8]. Endophytic fungal groups commonly establish a sole host specificity at the species level, which can be additionally encouraged by microclimatic conditions and microhabitat [9]. The relationship is chiefly stimulated by the endophyte fungi, yielding an overabundance of natural compounds as soon as endophytic fungi are cultured in the external environment of their ordinary hosts or environmental niches under in vitro test conditions [10].

Protocorm and seedling expansion is an important step in commercial orchid production, and its conservation is crucial tool for maintaining the genetic diversity of the orchid plant [11]. Orchid (*Dendrobium nobile* and *D. officinale*) protocorms initiating from the orchid seeds are typically very small, similar to dust, and deficient an endosperm. Subsequently, orchid seed incubation and seedling expansion require well-matched endophytic fungi to deliver the carbon, nutrients, and water to the seeds under usual plant conditions [3]. Consequently, an in vitro proliferation method could be a useful for the mass measure proliferation of these orchids for their marketization. The protocorms and seedlings rarely survive after relocation into nature from well mature sterile culture. The orchid protocorm may be reliant on appropriate endophytic fungi for seedling existence [12].

Most examinations of *Dendrobium* myco-endophytes (DMEs) focused on symbiotic in vitro practices using fungal endophyte strains obtained from fully-grown dendrobium roots, buds, stems, and leaves [3]. Understanding whether these endophytic fungi are pathogenic, conditional pathogenic, or non-pathogenic for the host plant is important. DMEs can be transmitted horizontal or vertically. Vertical transmission occurs when the seeds are contacted through the fungal endophytes and are transmitted to the host plant. Horizontal transmission involves the formation of exterior spores and their airborne dispersal infects many other hosts [13]. 

A steady state between the fungal pathogen and its host plant is achieved when the pathogen resides in equilibrium with the surrounding host tissues and causes little damage, which is also called least pathogenic. The virulence expression of the pathogen is dependent on the particular host environment. When the pathogen is isolated from an asymptomatic host and introduced into a new host, strong pathogenic reactions may be observed [14]. Commonly, fungal endophytes have functions ranging from latent pathogens to mutualistic symbionts. Reliant on the host genome type, some endophytic fungi may be pathogenic in stressed hosts, whereas they can be helpful in other conditions due to conditional pathogenic properties [15]. In the environment, orchids are chiefly dependent on these fungal endophytes for their nourishment and propagation along with the succeeding seedling (protocorm) phases. However, only imperfect quantitative approaches for assessing the *Dendrobium*–fungus connections at the protocorm and seedling phase are available at present, which places major constraints on understanding the host–endophyte relationships [16]. 

In this study, we focused on species-specific endophytic fungi pre-isolated from *D. nobile* plant parts, which were inoculated onto well-developed protocorms and seedlings of *D. nobile* and *D. officinale* for the evaluation of pathogenicity using in vitro inoculation and histopathological examination of infected tissues. The test endophytic fungus was re-isolated from the leaves tissues for examining Koch’s postulates. 

## 2. Results

### 2.1. Isolation and Molecular Identification of Endophytic Fungi

**Figure 1:**
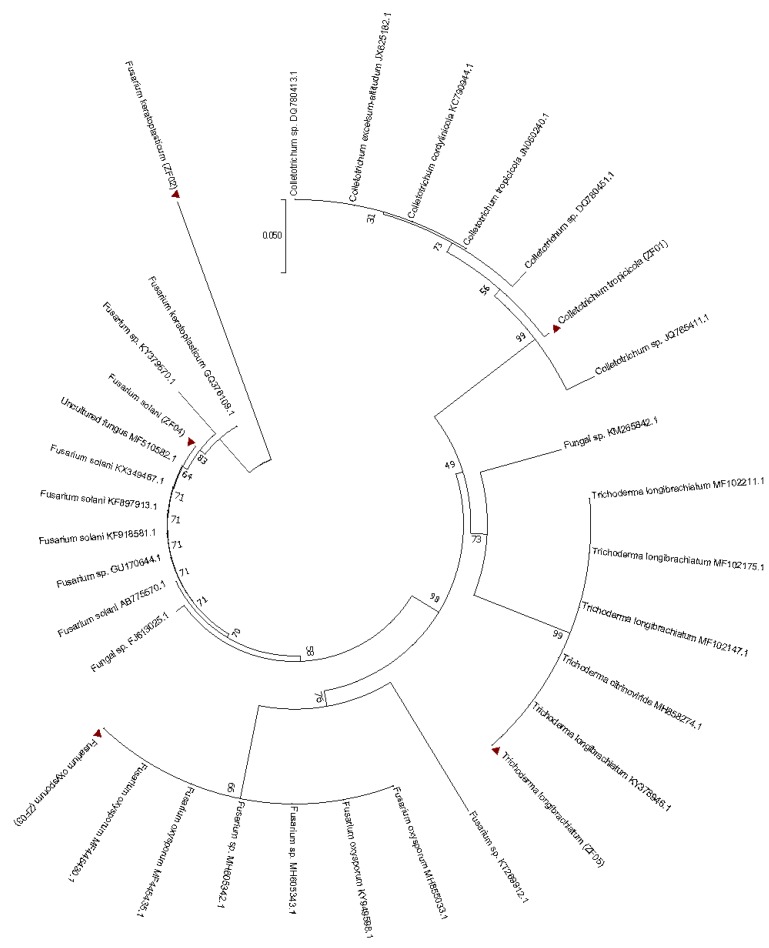
Phylogenetic identification of the endophytic fungi based on ITS4 and ITS5 regions of DNA sequences. The evolutionary detachments were calculated using the Kimura two-parameter method.

Five DME isolates were recovered (ZF01, ZF02, ZF03, ZF04, and ZF05) from leaf segments of the wild *D. nobile*. The colony morphological traits of the DME isolates, such as hyphal structures and spore arrangements, were used for their identification. The isolated endophytic fungal colony characteristics and microscopic characteristics findings were found as follows: (1) ZF01: fast grower (35 ± 0.2 mm), flat and buff growth surface, whitish color, reverse whitish to grey/orange, margin irregular mycelium with conidiogenous cells hyaline to pale brown, conidia hyaline with round ends, smooth-walled; (2) ZF02: moderate grower (16 ± 0.2 mm), colony flat velvety, white color with no diffusing pigment coloration, margin irregular, white rims, mycelium with microconidia oval and elongated and clavate, sickle-shaped conidia containing numerous septations, septate hyaline hyphae; (3) ZF03: fast grower (25 ± 0.2 mm), colony cottony white to light violet color, aerial white margins septate and hyaline, macroconidia slightly curved and thick with an attenuated apical cell, three to five septa, short aerial conidiophore, unbranched, microconidia non-septate, aerial conidia, ellipsoidal to cylindrical, slightly curved; (4) ZF04: fast grower (28 ± 0.2 mm), colony cottony white to light white with creamy to white-greyish and reverse light yellow color, irregular white margins with cream yellow edges with long and narrow macroconidia, cylindrical, dorsal and ventral surface parallel, three to five septa, phyalide long and thin; and (5) ZF05: fast grower (34 ± 0.2 mm), colony cottony off white to greenish, later green tufts sporulation, irregular white margins with very hyaline hyphae, long conidiophores, flask-shaped single phialides, small-walled ellipsoidal conidia, formed chlamydospores (smooth walled) in terminal or intercalary (Table S1). Molecular characterization was used to confirm isolate identification in which the rDNA (recombinant deoxyribose nucleic acid)-ITS (internal transcribed spacer) region was amplified. The sequences data were aligned using BLAST (basic local alignment search tool). The endophytic fungi were found to be the closest homologs of *Colletotrichum tropicicola* (ZF01), *Fusarium keratoplasticum* (ZF02), *Fusarium oxysporum* (ZF03), *Fusarium solani* (ZF04), and *Trichoderma longibrachiatum* (ZF05) (Figure 1). Results from BLAST were categorized for the isolated endophytes under ascomycota, which coincided with the morphological identification. These were represented by class Sordariomycetes, including isolates belonging to *Colletotrichum* and *Trichoderma*, and Hypocreales included isolates belonging to *Fusarium*. These were the most frequent and dominant endophytic fungi isolated from *D. nobile*.

### 2.2. Evaluation of Protocorm and Seedling Pathogenicity 

**Figure 2:**
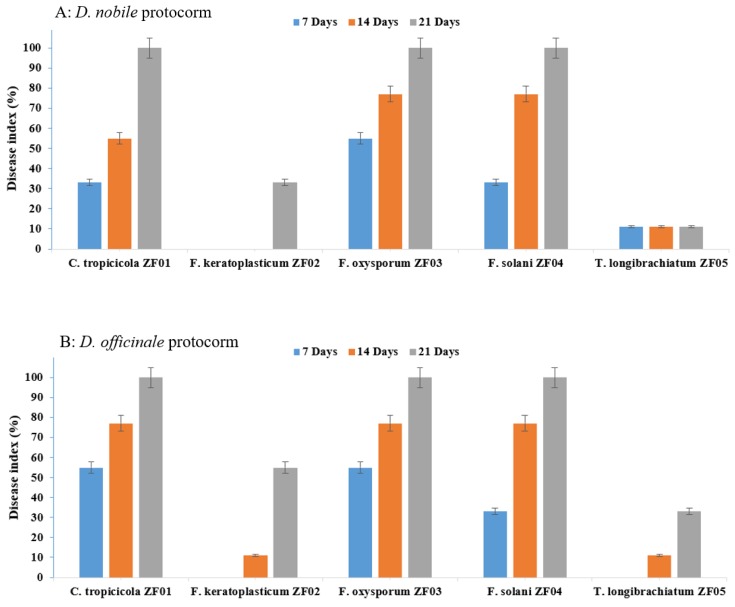
Co-culturing pathogenic effects on protocorm of (**A**) *D. nobile* and (**B**) *D. officinale* with endophytic fungi. Means values are significantly different at *p* = 0.05 according to Duncan’s multiple range test.

**Figure 3:**
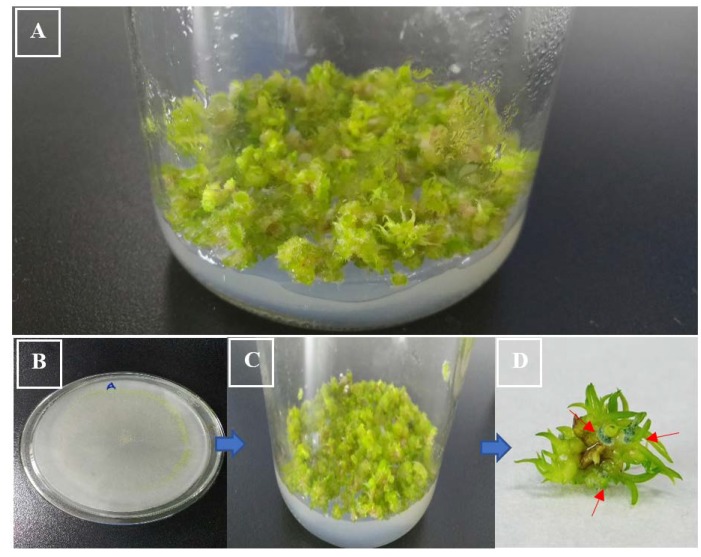
Least pathogenicity by *Trichoderma longibrachiatum* (ZF05) against the *D. nobile* protocorm after 21 days. (**A**) Control (*D. nobile* protocorm with water agar media); (**B**) pure culture of *Trichoderma longibrachiatum* (ZF05); (**C**) *D. nobile* protocorm with *Trichoderma longibrachiatum* (ZF05) after 21 days; (**D**) Single *D. nobile* protocorm with *Trichoderma longibrachiatum* (ZF05) growth indicated by the arrow.

The DME pathogenicity results with *D. nobile* and *D. officinale* protocorm revealed that *T. longibrachiatum* (ZF05) showed the least pathogenicity (11%) against the *D. nobile* protocorm after 7, 14, and 21 days, whereas it showed moderate pathogenic (33%) effects at 21 days of incubation against *D. officinale*. We found that most of the plant pathogens are latent pathogens or conditional pathogens, causing symptoms some weeks later when the plant defense mechanism has weakened. Many environmental factors function together to trigger the virulence factors or genes of the endophytic fungi. *T. longibrachiatum* (ZF05) started to change the color of the *D. officinale* protocorm from green to light yellowish at 14 days, which changed to brown to blackish at 21 days; whereas no color change symptoms were observed in the *D. nobile* protocorm. The second least pathogenic strain was *F. keratoplasticum* (ZF02), which was found to be non-pathogenic against *D. nobile* after 7 and 14 days of incubation, and least pathogenic (11%) against *D. officinale* after seven days of incubation, but showed moderate pathogenic effect after 21 days incubation on both *D. nobile* and *D. officinale*. The symptoms on the protocorms of *D. nobile* and *D. officinale* by *F. keratoplasticum* (ZF02) included the color of *D. officinale* protocorm starting to change from green to light yellowish at 14 days, then turning yellow at 21 days, whereas no color change symptoms were observed in the *D. nobile* protocorm due to *F. keratoplasticum* (ZF02). *F. oxysporum* (ZF03) and *F. solani* (ZF04) were moderate pathogens at seven days of incubation and strong pathogens (100%) at 21 days of incubation. *C. tropicicola* (ZF01) showed high pathogenicity (77–100%) effects against both *D. nobile* and *D. officinale* seedlings. *F. oxysporum* (ZF03) produced no symptoms on the protocorms of *D. nobile* and *D. officinale* at 7 and 14 days, indicating these are latent pathogens because they demonstrated high pathogenicity at 21 days and their symptoms included color change of the green protocorm to brownish for both *D. nobile* and *D. officinale*. *F. solani* (ZF04) produced moderate symptoms on the protocorms of both *D. nobile* and *D. officinale* by changing their green color to light reddish to brown in the initial days and then turned dark reddish-brown at 21 days. *C. tropicicola* (ZF01) significantly affected the protocorms of *D. nobile* and *D. officinale* by changing their green color to dark brown at 7, 14, and 21 days (Figure 2 and Figure 3).

**Figure 4:**
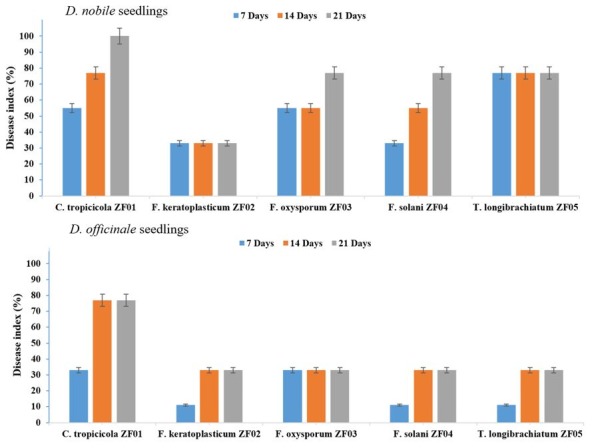
Co-culturing pathogenic effects of endophytic fungi on seedlings of *D. nobile* and *D. officinale*. Means values are significantly different at *p* = 0.05 according to Duncan’s multiple range test.

**Figure 5:**
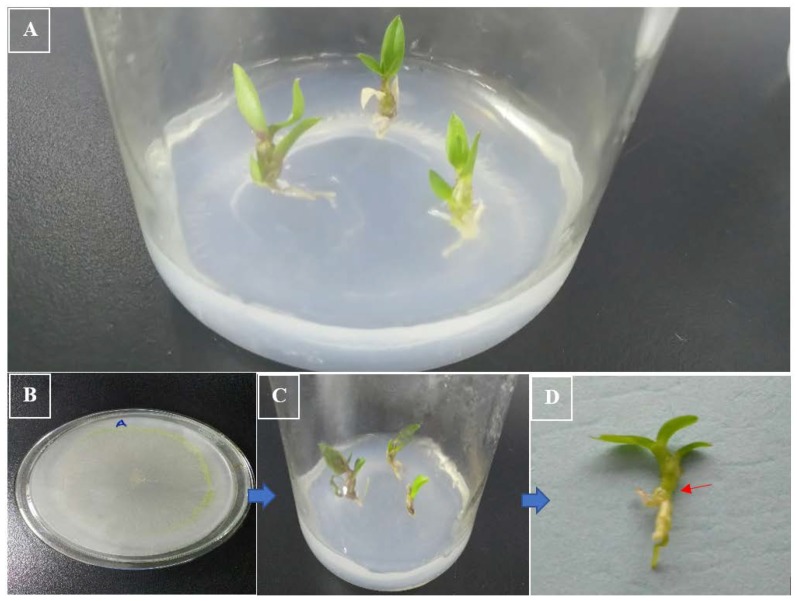
Moderate pathogenicity by *Trichoderma longibrachiatum* (ZF05) against the *D. officinale* seedling after 21 days. (**A**) Control (*D. officinale* seedling with water agar media); (**B**) pure culture of *T. longibrachiatum* (ZF05); (**C**) *D. officinale* seedling with *T. longibrachiatum* (ZF05) after 21 days; (**D**) Single *D. officinale* seedling with *T. longibrachiatum* (ZF05) growth indicated by the arrow.

The DME pathogenicity results for *D. nobile* and *D. officinale* seedling revealed that *T. longibrachiatum* (ZF05) produced the least pathogenic effects on *D. officinale* protocorm after seven days and increased gradually with increasing incubation period (at 14 days; 33% disease index), which was stable at 21 days (33% disease index). For *D. nobile*, the fungus showed highly pathogenic (77% disease index) effects from the start to the end of the incubation period. The second least pathogenic strains were *F. keratoplasticum* (ZF02) and *F. solani* (ZF04), which showed least pathogenic effects against *D. officinale,* whereas *F. keratoplasticum* (ZF02) and *F. oxysporum* (ZF03) showed stable moderate pathogenic (33%) effects against *D. nobile* and *D. officinale*, respectively, throughout the incubation period. *C. tropicicola* (ZF01) showed high pathogenicity (77–100%) against both *D. nobile* and *D. officinale* seedlings. The seedlings symptoms included a color change of *D. nobile* seedlings from green to black spots and brownish to blackish spots on the *D. officinale* seedlings with *T. longibrachiatum* (ZF05) at 7, 14, and 21 days. *F. keratoplasticum* (ZF02) produced symptoms on the *D. nobile* and *D. officinale* seedlings including color change of the *D. officinale* seedlings from green to yellowish at 7 days, then to light brown at 14 days, then to black at 21 days; similar symptoms were observed in the *D. nobile* seedlings with *F. keratoplasticum* (ZF02). *F. oxysporum* (ZF03) produced light brown color symptoms on the seedlings of *D. nobile* and *D. officinale* at 7 and 14 days, which later changed to brownish to yellowish. *F. solani* (ZF04) produced moderate symptoms on the seedlings by changing its green color to light yellowish to brown at 7 days, changing to dark brown at 14 days, then to blackish brown at 21 days on the seedlings of *D. nobile* and *D. officinale*. *C. tropicicola* (ZF01) produced significant symptoms on the seedlings of *D. nobile* and *D. officinale* by changing the seedling color from green to brown at 7, 14, and 21 days (Figure 4 and Figure 5).

### 2.3. Re-Isolation of Endophytic Fungi from Co-Culturing Seedling Tissues

**Figure 6:**
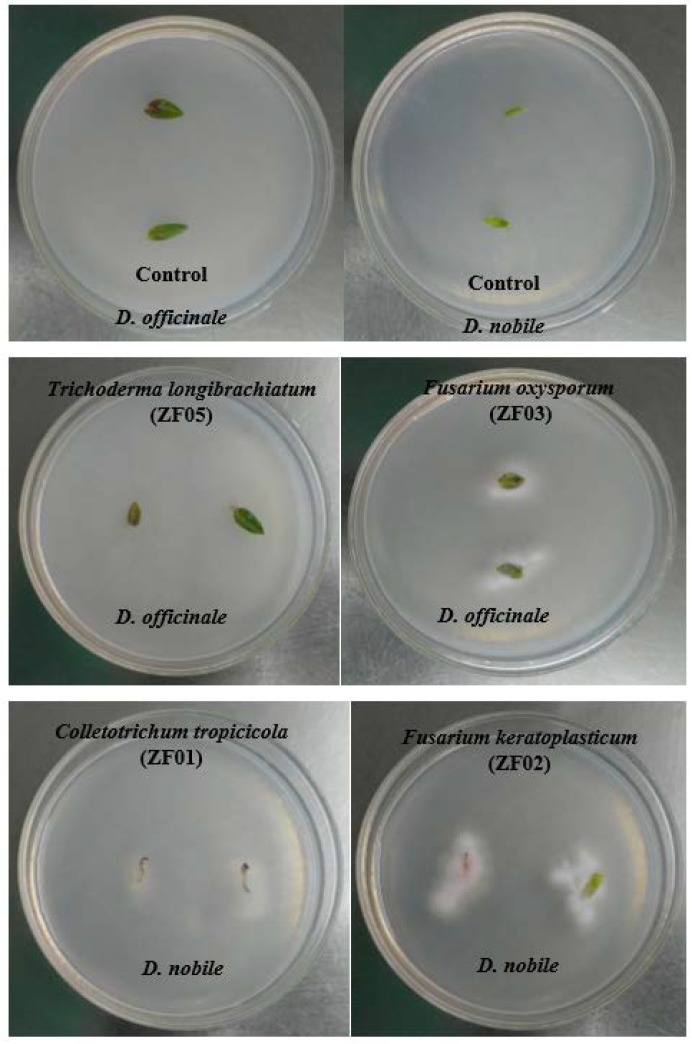
Re-isolation of test endophytic fungi from the uninoculated tissues of *D. nobile* and *D. officinale*.

After the coculturing incubation periods (21 days), the endophytic fungi were re-isolated from the plant tissues to confirm the presence of fungi inside the plant tissues by re-inoculating the samples into the growth media (PDA: potato dextrose agar media). The results showed that the same endophytic fungi were re-isolated from the uninoculated plant tissues, which were identified based on characteristics: *C. tropicicola* (ZF01), *F. keratoplasticum* (ZF02), *F. oxysporum* (ZF03), *F. solani* (ZF04), and *T. longibrachiatum* (ZF05). All isolated endophytic fungi were confirmed as the same fungi via microscopic examination as described in Table S1 and Figure 6.

### 2.4. Histological Examination of the Least Pathogenic Fungal Endophyte Seedling Samples

**Figure 7:**
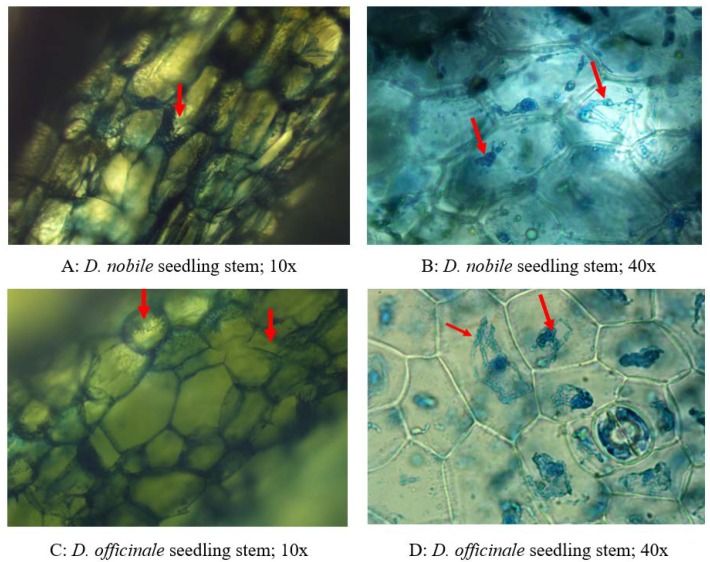
Histopathological examination of *D. nobile* and *D. officinale* stem infected by the least pathogenic test endophytic fungi, *T. longibrachiatum* (ZF05). (**A**,**B**) Transverse section of *D. nobile* stem showing the presence of *T. longibrachiatum* (ZF05). (**A**) *T. longibrachiatum* in epidermal region (10×). (**B**) Dense colonies of *T. longibrachiatum* (ZF05) in phloem with spores (40×). (**C**,**D**) Transverse section of *D. officinale* stem showing the presence of *T. longibrachiatum* (ZF05). (**C**) *T. longibrachiatum* (ZF05) in intercellular mycelia with less in epidermal region (10×). (**D**) Dense colonies of *T. longibrachiatum* (ZF05) in phloem with spores (40×).

The results showed that the thin sections stained with lactophenol cotton blue showed the presence of endophytic fungi in the intercellular spaces of *D. nobile* and *D. officinale* seedling tissues (Figure 7). The transverse section of *D. nobile* seedling stems at lower magnification showed dense blue *Trichoderma longibrachiatum* mycelia in the epidermis and mesophyll region (Figure 7A) and, at high magnification, we observed sprinkled colonies proximal to vascular bundles (Figure 7B). The transverse section of the *D. officinale* seedling stem at lower magnification showed *T. longibrachiatum* mycelia in the intercellular mycelia and a lower amount in epidermal region (Figure 7C); at higher magnification, we observed a large amount of *T. longibrachiatum* mycelium in the phloem region with slightly less in the epidermis (Figure 7D).

## 3. Discussion

*Dendrobium* is largest genera of Orchidaceae, with more than 1000 species globally [3]. *D. nobile* and *D. officinale* are a wild epiphytic orchid found in the tropical rain forests in China, especially in Guizhou province, China. The solitary and attractive inflorescence of these orchids is slightly exclusive and valued amongst orchid cultivators. Due to their high commercial demand, they are being removed from their usual habitat, placing them at high risk of becoming extinct. To preserve and reinstate threatened and rare orchid plants, these plants must be reintroduced with fungal co-culturing. Hence, some pure endophytic fungal strains that stimulate or decrease *Dendrobium* protocorm and seedling growth must be determined in vitro [17]. For this purpose, co-culturing will be essential for *D. nobile* and *D. officinale* success. Biosynthesis of many active compounds will be necessary, predominantly where specific fungal strains are vital for actively providing molecules to the protocorm with or without producing symptoms.

This study provides valuable information about the orchid bionetwork with endophytic fungi associated with *D. nobile* and *D. officinale* under laboratory environments. The in vitro pathogenicity evaluation process was used to effectively assess well-suited and species-specific endophytic fungi in terms of their symptoms and pathogenicity index for *D. nobile* and *D. officinale*. For the first time, we found that *T. longibrachiatum* (ZF05) produced the least pathogenic effects on *D. nobile* and *D. officinale*, which provided the plants with nutrition and helped them build an active defense mechanism to survive without the presence of any nutrients in the media. This phenomenon was observed as asymptomatic colonization due to the balanced antagonism between the host and the endophyte [13]. Endophytes also possess various virulence factors that are contradicted by host plant defense mechanisms. If endophyte virulence and host *Dendrobium* defense are well balanced, the relationship is avirulent and asymptomatic. This stage is only a transitory time where environmental influences play a key role in destabilizing the delicate equilibrium of antagonisms. [18]. Endophytic fungi can deliver appropriate carbon sources, amino acids, vitamins, and hormones that are important for seedling and protocorm development [19]. Khamchatra et al. [3] also stated that *Beauvaria* and *Fusarium* species are endophytic fungi, which we also recovered, that may play a role in the growth and survival of the plants like *D. friedericksianum*. Though grown in orchid stems and roots, the fungi may be non-casual and non-mycorrhizal endophytes. Many of these endophytes were reported in many white rot fungi and are incapable of phytopathogenicity [3]. In another study, Meng et al. [20] found that several fungal species recovered from some species of *Dendrobium* protocorms and seedlings have the ability to cause disease symptoms. Zi et al. [21] described *Epulohiza*, an anamorphic phase of *Tulasnella*, in the *Dendrobium* protocorms. Athipunyakom et al. [22] reported that *Trichosporiella multisporum* is present in *Paphiopedilum* roots. In an ecosystem, orchids depend on orchid fungal endophytes to provide needed nutrients for growth, a process termed an asymptomatic relationship. The symbiotic plant relationship, specifically in vitro approaches, is adopted because it enables higher growth rates and/or symbiotic seedlings and protocorms progress faster than without this relationship [11]. Although extensive information about the co-culturing expansion is not available, the process was endorsed as an effective process for improving the growth of many orchids [19].

From this study, we found that *C. tropicicola* (ZF01) is a highly pathogenic strain for the protocorms and seedlings of both *D. nobile* and *D. officinale*. *Dendrobium* endophyte contact might, in addition to balancing between defense and virulence, might more precisely control this complex contact [10]. Plant–pathogen association may be responsible for the growth conditions for plant disease. Because several fungal endophytes may be latent plant pathogens, certain inherent or environmental influences may prompt pathogenic effects [18]. Many fungal endophytes are silent/conditional pathogens, only resulting in disease as the plant ages or under stress conditions. Fungal endophytes accomplish asymptomatic colonization through a stable neutral antagonism between the fungal virulence and its response to the plant defense mechanism [23]. We hypothesized from our research that *T. longibrachiatum* (ZF05) mycelium may migrate from inoculated to uninoculated tissues of host plants. The histological image of *D. nobile* and *D. officinale* seedlings clarified that the fungus infects tissues. This paper is the first report on the cross-transmission of *T. longibrachiatum* (ZF05) from inoculated tissues to uninoculated tissues confirmed by re-isolated from host plant segments that fulfilled the Koch postulates. *Colletotrichum, Fusarium*, and *Trichoderma* include morphologically similar taxa that are commonly found as endophytes, saprobes, and plant pathogens [13,14,15]. 

As endophytes exist in within plant tissues and endlessly network with their host tissues, fungal endophytes may be linked intracellularly, which is responsible for the cross-transmission of the fungus into the new cells. Along this line, we strongly suggest that endophytic fungus is re-isolated from the uninoculated infected segments of the *D. nobile* and *D. officinale* seedlings. This means that these endophytic fungi are cross-transmitted intracellularly from one cell to another healthy cell, creating comprehensive endophytic molecular connections, cross-species appearance, and on/off switching of the compulsory gene cascades that constantly yield a chosen molecule [10]. 

## 4. Materials and Methods

### 4.1. Isolation of Endophytic Fungal Strains 

Endophytic fungi were isolated from leaves parts. Five healthy wild *D. nobile* plants were collected (from agricultural farm single location) from Jinshishi, Chishui, Guizhou, China and processed in the Bioresource Institute for Health Utilization, Zunyi Medical University, Zunyi, China, and then inoculated aseptically into PDA media. The samples were processed according to a method modified from Novotná et al. [8]. All the leaves segments of wild *D. nobile* were used for surface sterilization processing. All the leaves samples were washed with tap water to remove the dirt spots and connective tissue of stems. Thereafter, the leaves were washed with tap water to further clean the surface. After cleaning, all the plant samples were placed into laminar air flow and exposed to UV rays for 20 min. Under sterilized conditions in the laminar air flow, the samples (1.5 cm pieces) were inoculated onto PDA media plates. The sample plates were placed in a fungal incubator at 25 °C for 5 days. After 5 day of incubation, the fungal hyphal tips emerging from the samples were recovered and purified by sub-culturing from the grown hyphal tips.

### 4.2. Morphological and Molecular Identification of Plant Endophytic Fungi

The endophytic fungal genus was identified using lactophenol cotton blue staining according to the protocol followed by Barnett and Hunter [24] and Ellis [25]. The DNA was extracted from approximately 100 mg of fungal mycelia using the DNAsecure Plant Kit according to the manufacturer’s instructions (Tianjin Biotech Co. Ltd., Beijing, China). PCR was performed using a standard protocol [26]. The reactions were prepared in 25 μL volumes constituting 2.0 μL primer, 12.5 μL 2× TsingKe (Blue) Master Mix (TSINGKE Biological Technology Co., Ltd, Beijing, China), 9.5 μL RNase-free distilled water, and template DNA (2 μL). A non-template tube was used as a negative control during PCR amplification to monitor purity. Amplifications were completed using a PCR thermal cycler equipment (Bio-Rad C1000 Thermal Cycler, Minnesota, USA). PCR cycling was performed using the conditions and primer sequences [27] presented in Table S2. 

### 4.3. Electrophoresis of PCR Products

The PCR samples were analyzed using electrophoresis on a 1% (w/v) agarose gel (Agarose G-10, Gene Company Ltd., Hong Kong, China) [28]. A DNA marker (100 to 1000 bp molecular weights) (Beijing TransGen Biotech Co. Ltd., Beijing, China) was added to the first well of the gel. Electrophoresis was conducted in a horizontal electrophoresis system (Bio-Rad PowerPac^TM^ Basic, Henderson, Singapore) for 30 min at 120 V and 250 mA using 1× TAE (Tris-acetate-EDTA) buffer. The gel was marked with ethidium bromide (0.1 μg/mL). A Molecular Imager Gel Doc^TM^ XR+ with Image Lab Software system (Bio-Rad Gel Doc^TM^ XR+ Imaging System, Hercules, CA, United States) was used to capture images using Image Lab software (version 4.1).

### 4.4. Sequence Analysis of PCR Products

The amplified ITS regions of PCR samples were sequenced by Tsingke, Beijing, China. Sequence data were subjected to BLAST analysis on the NCBI (national center for biotechnology information) web tool to validate the characteristics of the amplified sequences along with isolates. All the sequences have been deposited in the GenBank under accession numbers i.e., *Colletotrichum tropicicola* ZF01: MN826680; *Fusarium keratoplasticum* ZF02: MN832906; *Fusarium oxysporum* ZF03: MN826681; *Fusarium solani* ZF04: MN826682; and *Trichoderma longibrachiatum* ZF05: MN826683. MEGA 7.0 software was used for phylogeny tree study.

### 4.5. Collected Samples (Protocorm and Seedling) and Sample Processing

**Figure 8:**
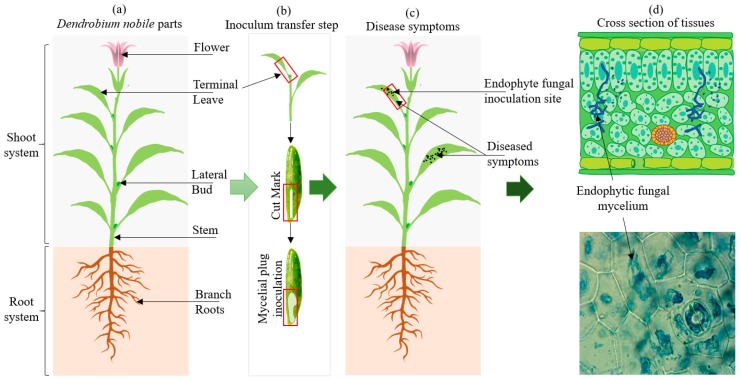
Different phases of coculturing experiment for pathogenicity evaluation. (**a**) Healthy seedlings; (**b**) cut marks at the top leaf and inoculation of mycelial plug of endophytic fungi; (**c**) symptoms developed for pathogenicity evaluation; and (**d**) histopathological examination of infected tissues.

Water agar (0.65% w/v) was used as media and relocated into the glass bottles for the coculturing experiments. No additional nutriments were added into the media; only twofold distilled water and agar were used. *D. nobile* and *D. officinale* (both protocorms aged 4 months) and seedlings (aged 8 months) were obtained from the Bioresource Institute for Healthy Utilization (BIHU), Zunyi Medical University (ZMU; Zunyi, Guizhou, China) for experiments and authenticated per previous work [29,30,31,32]. After the sterilization of the media bottles, the protocorms of the *D. nobile* and *D. officinale* were inoculated into separate bottles under aseptic conditions. For seedlings, we cut marks into the top leaf of the seedlings where it joined then stem using a sterilized scalpel blade over sterilized paper, as shown in Figure 8. Three seedlings were transferred into each bottle under aseptic conditions for pathogenicity and symptoms evaluation.

### 4.6. In Vitro Inoculation for Seedling Pathogenicity Assay

For pathogenicity experiments, the identified endophytic fungal mycelium was used for the pathogenicity evaluation. For protocorms, 0.5-cm fungal discs were transferred into the protocorm bottles of *D. nobile* and *D. officinale*. The control was set in triplicates under the same conditions without added any endophytic fungal cultures. After the inoculation, the test co-culturing bottles were transferred into the plant growth chamber instrument and maintained at 25 °C along with a 14/10 h light/dark cycle with 2000 lux light intensity. All the test bottles were examined in 7-day intervals up to 21 days. For *D. nobile* and *D. officinale* seedlings, the contents of endophytic fungal flask cultures were filtered to separate the mycelium suspension. The mycelium was concentrated and injected onto cut marks at terminal leaves at the link to the stem. The sterilized cotton plug served as a control for seedlings co-culturing pathogenicity and symptoms evaluation experiments. The disease index was measured using the disease scale and disease index formula. The scale of the disease index is described as follows: 0 indicates no disease (non-pathogens), 1 indicates small spots < 1% (pin brown spots: fewest pathogens), 3 indicates spots (1–11%: least pathogens), 5 indicates spots with 12–25% coverage (moderate pathogens), 7 indicates circular spots (26–55% coverage: moderate pathogens), and 9 indicates circular to irregular spots (>75% coverage: highly pathogenic) [33].

$$Disease\;index\;percentage=\frac{Sum\;of\;disease\;rating}{Total\;number\;of\;rating\;\times \;Maximum\;disease\;grade}\;\times \;100\%$$

### 4.7. Re-Isolation and Identification of Endophytic Fungi from Seedlings

All the samples (seedlings) were washed with sterilized distilled water to eliminate surface fungal growth. Thereafter, the seedlings were placed into laminar air flow and exposed UV rays for 20 min to eliminate surface fungi. After UV exposure, 75% alcohol was added into the sample bottles for 30 s and mixed using gentle shaking, which was then washed twice with sterilized distilled water under laminar airflow. Thereafter, HgCl_2_ was immersed into the glass bottle for 3 min with continuous shaking. The HgCl_2_ water was removed after 3 min and then the samples were washed 3 times using sterilized distilled water. Brown paper sterilized in a hot air oven at 160 °C for 1.5 h was used for cutting the samples into smaller pieces. The samples (seedling) were moved onto the potato dextrose agar media plates. Two pieces of samples were transferred onto each Petri plate with the surface touching the media. The plates were incubated for five days at 25 °C in the incubator. After the fifth day of incubation, fungal hyphal tips emerging from the samples were recovered and purified using sub-culturing from growing hyphal tips. The growth, colony properties, and microscopic features of DMEs were recorded from the strains grown on the PDA media [34,35]. Microscopic details were obtained via a slide culture practice. Fungal hyphae stained with lactophenol cotton blue were assessed microscopically using light microscopy (Nikon Eclipse E200MV R with DS-1600 Panasonic CMOS Sensor, Nikon Corporation, Tokyo, Japan).

### 4.8. Histological Examination of Least Pathogenic Fungal Endophyte Samples

Infected *D. nobile* and *D. officinale* samples were used for visual histopathological assessment of endophytic fungi by staining the infected tissues using a method modified from Ding et al. [36]. Infected seedling segments were stained using lactophenol cotton blue to identify the tissue infected with endophytic fungi. The distribution of fungal endophytes and their localization was studied using a microscope (Nikon Eclipse E200, Model Eclipse E200MV R, Nikon Corporation, Tokyo, Japan) with DS-1600 Panasonic CMOS sensor. The endophytic fungal colonies were observed as blue after staining with lactophenol cotton blue in vascular bundles and cortex region of *D. nobile* and *D. officinale* leaves and stems. Photographs were captured under different magnifications (10× and 40×).

### 4.9. Statistical Analysis

Statistical calculations were completed using SPSS11 software (USA) and the Fisher’s least significant difference procedure. Correlations of disease index caused by *C. tropicicola* (ZF01), *F. keratoplasticum* (ZF02), *F. oxysporum* (ZF03), *F. solani* (ZF04), and *T. longibrachiatum* (ZF05) were calculated using SAS CORR procedure.

## 5. Conclusions

In this study, we evaluated fungal pathogenicity and colonization inside plant tissues under in vitro conditions. Firstly, *T. longibrachiatum* (ZF05) was found to be the least pathogenic or a conditional pathogen that supports the development of the *D. nobile* protocorms and *D. officinale* seedlings without the presence of any nutrients in the media. *C. tropicicola* (ZF01) is a highly pathogenic strain, responsible for the host death. We concluded that endophytic fungi were cross-transmitted from host plant inoculated to uninoculated cells, which was confirmed by histopathological examination and re-isolation of the same endophytic fungi from uninoculated plant tissues. Future investigations should determine what role, if any, the plant host specificity plays in the interior plant passage and differential tissues establishment by test fungal endophytes. How fungi are able to precisely move within tissue (host plant) should also be examined. The symbiotic seedling and protocorm growth are advantageous and expedient method to improve orchid growth under the experimental conditions and could help with the reintroduction of *Dendrobium* orchids to the natural environment.

## Supplementary Materials

The following are available online at https://www.mdpi.com/1422-0067/21/1/316/s1.

## Abbreviations

DME	Dendrobium myco-endophytes
PDA	Potato dextrose agar
DNA	Deoxyribose nucleic acid
PCR	Polymerase chain reaction
ITS	Internal transcribed spacer
NCBI	National centre for biological information
BLAST	Basic local alignment search tool
UV	Ultra Violet
HgCl2	Mercury chloride
