# Supplementary material for: Molecular Identification of Endophytic Fungi and Their Pathogenicity Evaluation Against Dendrobium nobile and Dendrobium officinale
Source: Int J Mol Sci. 2020 Jan 2;21(1):316. doi: 10.3390/ijms21010316 (PMC6982089; doi:10.3390/ijms21010316)
Supplement: Supplementary file 1 [file ijms-21-00316-s001.pdf]

Table S1. Growth conditions, and colony and morphological characteristics of isolated from wild *Dendrobium nobile* and re-isolated endophytic fungus after pathogenicity experiment.

Isolate name	Code	Host part for isolation	Host part for re-isolation	Media	Growth conditions	Colony diameter (mm, after 5 days)	Key colony characteristics			Key microscopic characteristics
							Surface	Color	Margin	
Colletotrichum tropicicola	ZF01	Wild *D. nobile* leaves	*D. nobile* and *D. officinale* seedling leaves	PDA	25 °C for 5 days	32 ± 0.2	Flat, white, buff cottony	Whitish to grey/ orange	Irregular margin mycelium	Sclerotia absent, conidiogenous cells hyaline to pale brown, conidia hyaline with round ends, smooth-walled
Fusarium keratoplasticum	ZF02	Wild *D. nobile* leaves				16 ± 0.2	Flat velvety	White with no diffusing pigment color	Irregular white rims mycelia	Microconidia oval and elongated and clavate, sickle-shaped conidia containing numerous septations, septate hyaline hyphae
Fusarium oxysporum	ZF03	Wild *D. nobile* leaves				25 ± 0.2	Cottony white to light violet colony	White to white-violet	Aerial white margins	Septate and hyaline, Macroconidia slightly curved and thick with an attenuated apical cell, 3 to 5 septa, short aerial conidiophores, unbranched, microconidia non-septate, aerial conidia, ellipsoidal to cylindrical, slightly curved,
Fusarium solani	ZF04	Wild *D. nobile* leaves				28 ± 0.2	Cottony white to light yellow colony	White with creamy to white-greyish, light yellow color	Irregular white margins with cream yellow edges	Long and narrow macroconidia, cylindrical, dorsal and ventral surface parallel, 3 to 5 septa, phyalide long and thin
Trichoderma longibrachiatum	ZF05	Wild *D. nobile* leaves				34 ± 0.2	Cottony off white to greenish	Off white color, later green tufts sporulation	Irregular white margins	Very hyaline hyphae, long conidiophores, flask-shaped single phialides, small-walled ellipsoidal conidia, formed chlamydospores (smooth

walled) in terminal or intercalary

Note: Values are presented as means ± SD; PDA, potato dextrose agar

Table S2. Primer sequences and PCR conditions for amplification of internal transcribed spacer region.

Primer	Length	Sequence (5′ to 3′)	PCR conditions
ITS 4	20	TCCTCCGCTTATTGATATGC	95°C for 30 s
ITS 5	22	GGAAGTAAAAGTCGTAACAAGG	58°C for 30 s 35 Cycles
			72°C for 40 s

Note: s: Seconds; ITS: internal transcribed spacer
